# Molecular and morphological characteristics of *Trichuris tenuis* from South American Camelids bred in Europe

**DOI:** 10.3389/fvets.2026.1832113

**Published:** 2026-05-07

**Authors:** Simona Rejnková, Ondřej Daněk, Ondřej Máca, David Modrý, Václava Hrabětová, Vincenzo Veneziano, Elisa Castaldo, Mikuláš Oros, Jiří Patoka, Zuzana Čadková, Ivana Jankovská, Silvie Neradilová, Radim Kotrba, Martin Ptáček, Iva Langrová

**Affiliations:** 1 Department of Zoology and Fisheries, Faculty of Agrobiology, Food and Natural Resources, Czech University of Life Science Prague, Prague, Czechia; 2 Department of Veterinary Sciences, Faculty of Agrobiology, Food, and Natural Resources, Czech University of Life Science Prague, Prague, Czechia; 3 Department of Pathology and Parasitology, State Veterinary Institute Prague, Prague, Czechia; 4 Department of Veterinary Medicine and Animal Production, University of Naples Federico II, Napoli, Italy; 5 Institute of Parasitology SAS, Košice, Slovakia; 6 Department of Animal Physiology, Institute of Biology and Ecology, P. J. Šafárik University, Košice, Slovakia; 7 Department of Wildlife and Animal Sciences, Faculty of Tropical Agrisciences, Czech University of Life Science Prague, Prague, Czechia; 8 Department of Animal Science, Faculty of Agrobiology, Food, and Natural Resources, Czech University of Life Science Prague, Prague, Czechia

**Keywords:** alpacas, COI, guanacos, llamas, parasites, rDNA, South American Camelids (SACs), *Trichuris tenuis*

## Abstract

**Background:**

The intestinal nematode *Trichuris tenuis* Chandler, 1930 is an important parasite associated with South American Camelids (SAC). To date, the species *T. tenuis* has been characterized solely based on morphology. The aim of this study was to comprehensively characterize these nematodes using an integrative taxonomic approach.

**Methods:**

Morphological, biometrical, and molecular analyses of *T. tenuis* specimens collected from alpacas (*Lama pacos*), llamas (*Lama glama*), and guanacos (*Lama guanicoe*) were performed. A total of 58 nematodes originating from three European farms and one zoo (located in Czech Republic and Italy) were subjected to morphological and biometric evaluation. For original DNA analysis, three markers were selected: a partial sequence of the nuclear 18S rRNA (18S) gene, both nuclear internal transcribed spacer regions (ITSs; encompassing a partial sequence of the 18S rRNA gene, ITS1, 5.8S, ITS2, and a partial sequence of the 28S rRNA gene), and a partial mitochondrial gene encoding cytochrome c oxidase subunit 1 (COI).

**Results:**

Males exhibited an extremely long cloaca with a complex, unique course, as well as a long, slender spicule, measuring 4.7–5.6 mm. Additionally, the junction between the ejaculatory duct and the vas efferens formed a distinct muscular constriction. Females exhibited an uneverted aspinous vulva and a highly convoluted vagina containing a unique egg chamber. A subset of adult worms was characterized using three molecular markers, which yielded 30 unique sequences: two variants for 18S, 14 variants for the ITS regions, and 13 variants for COI. Phylogenetic trees constructed for all DNA markers placed *T. tenuis* close to a well-defined clade of trichurid species parasitic to domestic ruminants.

**Conclusion:**

Integrative morphological, molecular, and phylogenetic analysis indicated that this species is both morphologically and molecularly distinct from its congeners. Furthermore, the evidence suggests that it exclusively infects SACs and potentially other camelids within captive populations worldwide. Similar integrative approaches are necessary for the taxonomic revision of the genus *Trichuris* and should be applied more frequently in future taxonomic research.

## Introduction

Parasitic nematodes in the genus *Trichuris* Roederer, 1761 (Nematoda, Trichuridae), commonly known as whipworms, are important inhabitants of the caudal part of mammalian digestive system with a range of interactions with host health and disease. These relatively large nematodes are deeply embedded with their whip-like cranial part into intestinal mucosa of the large intestine. There is good evidence of immunomodulatory functions of whipworm infections, that triggers a complex interplay between parasites and host health, involving also impact on the balance of the gut microbiota, both animals and humans (1–3). The whipworm infections are recognized as severe clinical situation altering host health, leading to death case of in massive infection (4). At the same time, whipworms are key model nematodes in studies addressing potential of whipworm-induced immunomodulation for treating chronic inflammatory-associated diseases (5, 6). Compared to *Trichuris* in rodents and primates (*T. muris* and *T. trichiura*), less attention is paid to the whipworms affecting herbivores.

The importance of trichuriasis for farm animal husbandry is obvious from classical as well as modern studies (7, 8), however, in ungulates, the understanding of *Trichuris*-host interactions is complicated also by the diversity and taxonomic complexity of parasite itself. The genus *Trichuris* currently involves more than 80 described species (9, 10), but its diversity is apparently higher, calling for integrative taxonomy approaches. Whipworms are frequently found in all species of camels (11–14) as well as in South American Camelids (SAC), both inside and outside areas of native distribution range (15–20).

The domesticated llamas and alpacas are becoming increasingly popular worldwide, especially as a therapy, companion and hobby animals (21–24) and range of studies described importance of endoparasitoses in these settings (16, 25–27). Llamas (*Lama glama*) and alpacas (*Vicugna pacos*) are domestic animals domesticated more than 5,000 years ago in the Andes Mountains (28), while the guanaco (*Lama guanicoe*) and vicuña (*Vicugna vicugna*) are wild representatives of the South American Camelids distributed across a wide range of environments in Peru, Bolivia, Chile, Paraguay, and Argentina.

Our understanding of situation of whipworms in Old and New World camelids resembles the knowledge gaps in other herbivores. Eggs of trichurids are commonly found in feces of SACs worldwide, however, in most cases, they are identified as *Trichuris* spp. (15, 16, 18, 20, 29) or as *T. tenuis* (17, 30).

Compared to studies relying on morphology, molecular data on *Trichuris* spp. associated with camelids are even more scarce. Only three camelid species are represented as hosts of *Trichuris* sequences in GenBank—Bactrian camel (*Camelus bactrianus*), dromedary camel (*Camelus dromedarius*), and alpaca. Dromedary camel is the only host where *Trichuris* sequences were obtained in the geographic range of the host species (North Africa and Middle East). Majority of these sequences are named as *T. globulosa*, in some cases with corresponding morphology (31), remaining sequences are referred to as *Trichuris* sp. (12). On the other hand, all molecular data on *Trichuris* from Bactrian camel and alpaca come from animals kept in zoos outside the geographic range of both species and are described as *Trichuris* sp. without any connection to morphology of the worms (32–35). To resolve persisting taxonomic gaps related to this group of parasites of high veterinary importance, our study combines morphological, molecular and phylogenetic characterization of whipworms collected from llamas, alpacas and guanacos from four different localities in Europe.

## Materials and methods

### Sample collection

Nematode specimens were collected from four sites, comprising three farms and one zoo over period 2023–2025. One farm is located in Italy, while the remaining farms and the zoo are situated in the Czech Republic. The first Czech farm, located in Mladá Boleslav (designated F1CZ), and the Italian farm, situated in Ercolano, in the province of Naples (designated F3It), both housed alpacas (*V. pacos*). Additional samples were obtained from Tábor Zoo, which maintains a population of llamas (*L. glama*). More recently, nematodes were also collected from guanacos (*L. guanicoe*) raised on the Farm of the Czech University of Life Sciences Prague (designated F2CZ).

All experimental procedures and animal husbandry practices were approved by the Institutional Animal Care and Use Committee of the Czech University of Life Sciences.

Adult trichurid nematodes were recovered either directly from the intestines of deceased hosts (F3It: *n* = 2 animals, F1CZ: *n* = 1 animal) or from the feces of dewormed animals (Tábor Zoo: *n* = 2 animals and F2CZ: *n* = 5 animals). Fecal samples were collected 24–36 h post-treatment and subsequently passed through 500-μm-mesh sieves.

### Morphological analysis

Each nematode specimen was rinsed twice in a saline solution to remove debris and subsequently fixed in 70% ethanol (samples from F1CZ, F3It, and Tábor Zoo). For morphological examination, specimens were cleared in glycerin, lactophenol, or phenol (36–38). Structural characteristics were examined under optical light microscopy at magnifications ranging ×5 to ×400 (Nikon Ni-E and Software NIS-Elements). Morphological measurements were compared with published descriptions (19, 38) to identify specimens to species level. Line drawings were made using a Leica DM 5000B light microscope (Leica Microsystems, Wetzlar, Germany).

The samples from the farm of the Czech University of Life Sciences (F2CZ) were examined immediately after collection and stored in saline solution for 6 days before fixation. After morphological examination and measurement, these trichurid specimens were fixed in 70% ethanol.

### Molecular and phylogenetic studies

Molecular data were obtained from 2–10 individual whipworms of each sex per site (except Tábor Zoo, where only females were obtained). Genomic DNA was extracted from the cranial region of selected worms using the NucleoSpin Tissue XS Kit (Macherey-Nagel, Germany) following the manufacturer’s protocol, using a final elution buffer volume of 20 μL. The obtained DNA was stored at −20 °C prior to further processing.

To molecularly characterize the specimens, three markers were selected: a partial sequence of the nuclear 18S rRNA (18S) gene, both nuclear internal transcribed spacer regions (ITSs; encompassing a partial sequence of the 18S rRNA gene, ITS1, 5.8S, ITS2, and a partial sequence of the 28S rRNA gene), and a partial sequence of the mitochondrial gene encoding cytochrome c oxidase subunit 1 (COI). All PCRs were performed in a total volume of 25 μL, consisting of 12.5 μL of 2 × PCRBIO Taq Mix Red (PCR Biosystems, United Kingdom), 0.4 μM of each primer, and 2 μL of template DNA; PCR-grade H_2_O served as a negative control. Details regarding these reactions are provided in Table 1. The resulting amplicons were separated by electrophoresis on a 1.5% agarose gel stained with Midori Green Advance (Nippon Genetics Europe, Germany) and visualized under UV light. All products exhibiting amplicons of the expected size were purified using the Gel/PCR DNA Fragments Kit (Geneaid, Taiwan). Purified PCR products were Sanger sequenced in both directions using the amplification primers by Macrogen Capillary Electrophoresis Sequencing services (Macrogen Europe, Netherlands). The sequences obtained were processed with Geneious Prime software and compared with those available in the GenBank database using the Basic Local Alignment Search Tool (BLAST).

**Table 1:** Details of the PCR protocols used in this study.

PCR	Primer sequence (5′–3′)	Annealing (°C)	Product length	Reference
18S	18S 965 (GGCGATCAGATACCGCCCTAGTT)	55	710 bp	Powers et al. (64)
	18S 1573R (TACAAAGGGCAGGGACGTAAT)			
COI	TriF3 (CTGCTAATCACAAAAAAATYGG)	52	1,272 bp	This study
	Trichuris_cox1_R (GAAAGTGTTGGGGYAKAAAAGTTA)			Wasimuddin et al. (65)
ITSs	NC5 (GTAGGTGAACCTGCGGAAGGATCATT)	57	1,200 bp	Zhu et al. (66)
	NC2 (TTAGTTTCTTTTCCTCCGCT)			Gasser et al. (67)

To evaluate the obtained sequences, a phylogenetic tree was generated for each molecular marker. All trees were constructed using either a subset (ITSs, COI) or all (18S) unique GenBank sequences exceeding 300 bp in length. The clade containing *T. navonae*, *T. pardinasi*, and related lineages was excluded, as initial data indicated that these taxa were too divergent from other *Trichuris* species. All phylogenies were inferred using IQ-TREE v.1.6.12 (39), with the best-fit evolution model selected based on the Bayesian information criterion, computed and implemented in ModelFinder (40). Branch support was rigorously assessed using ultrafast bootstrap (UFBoot) approximation (41) and the Shimodaira-Hasegawa-like approximate likelihood ratio test (SH-aLRT) (42). The resulting trees were visualized in FigTree v.1.4.4 and finalized using Inkscape v.1.3. Details of individual phylogenies are provided in the respective figure legends.

## Results

In this study, 58 identified as *Trichuris tenuis* collected from llamas (*L. glama*), alpacas (*V. pacos*), and guanacos (*L. guanicoe*) were examined using morphometric and molecular methods. All biometrical parameters of Trichuris tenuis isolated from SACs from three farms and a zoo are shown in Supplementary Table 1.

### Morphological characteristics

#### Females

**Figure 1:**
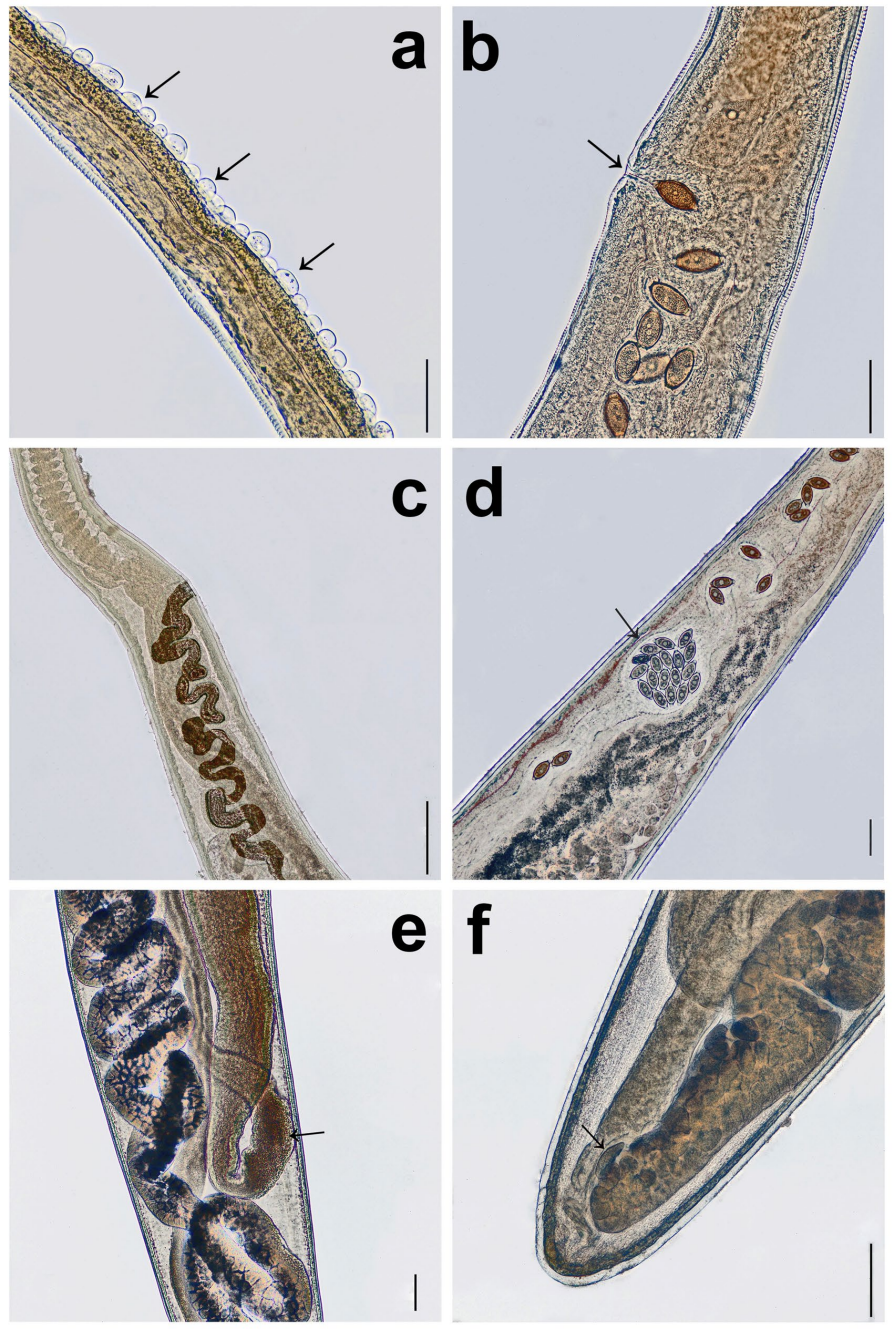
Females of *Trichuris tenuis*. **(a)** Bacillary band (arrows). **(b)** Uneverted aspinous vulva (arrow). **(c)** Highly convoluted vagina (arrow). **(d)** Egg chamber (arrow). **(e)** Receptaculum seminis (arrow) located behind the uterus. **(f)** Ovary with a ligament-like structure (arrow). Scale bars: **(a)** = 50 μm; **(b–f)** = 100 μm.

**Figure 2:**
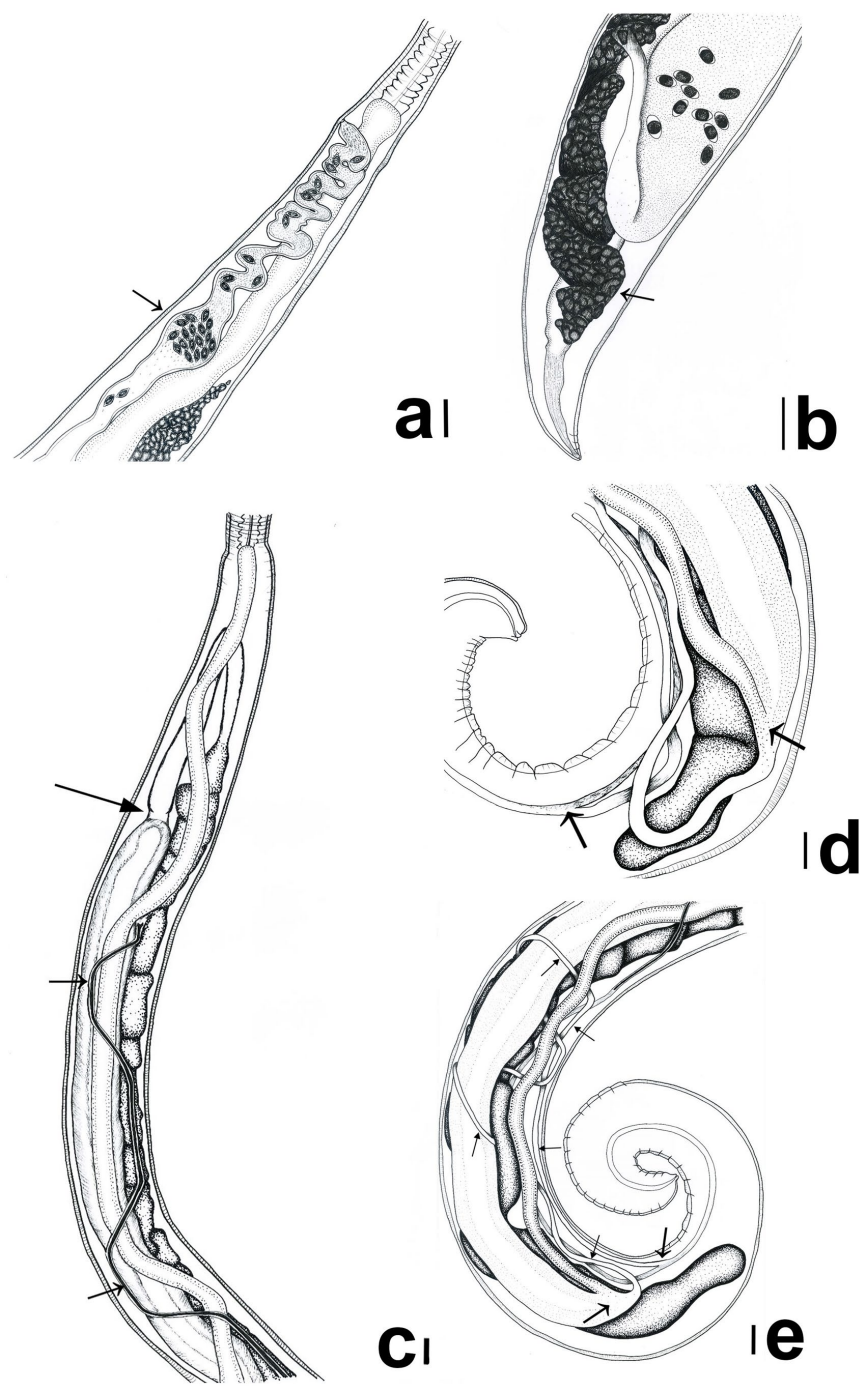
*Trichuris tenuis*. **(a)** Female: Highly convoluted vagina containing an egg chamber (arrow). **(b)** Female: Posterior end of female, ovary (arrow). **(c)** Male: Long, slender, winding spicule (thin arrows), and the junction between the ejaculatory duct and the vas efferens (thick arrow). **(d)** Male: The intestine and the ejaculatory duct converge to form the cloaca (arrow on the right), which then opens into the spicular sheath (lower-left arrow). **(e)** Male: The origin of the cloaca and its opening into the spicular sheath (thick arrows) and the whole course of the very long and thin cloaca through the body (thin arrows). Scale bars: **(a–e)** = 100 μm.

The total body length ranges from 28.5 to 43.5 mm, with the slender anterior portion measuring 18.4–32.1 mm. The thicker posterior portion is slightly curved and measures 4.7–17.1 mm in length. At the junction of the esophagus and intestine, the anterior portion is 103–277 μm wide, whereas the posterior portion reaches a width of 344–968 μm. Unlike males, females lack caudal papillae. The bacillary band (Figure 1a) originates 124–804 μm from the anterior extremity and extends 0.5–1.9 mm posteriorly. An uneverted, aspinous vulva (Figure 1b) is positioned either slightly posterior to or at the esophageal-intestinal junction. The vagina is highly convoluted throughout its length and features an egg chamber (122–375 μm wide) located 873–1990 μm from the vulva (Figures 1d, 2a). Posterior to this chamber, the size of the vaginal fold decreases slightly. Papillary spines are present in the proximal quarter of the vagina. The straight-line distance from the vulva to the vaginal-uterine junction measures 1.1–3.7 mm. The uterus, 5.5–7.8 mm in length, extends posteriorly with a maximum width of 208–738 μm, filling most of the posterior body before narrowing and turning anteriorly to become the oviduct. The receptaculum seminis is situated immediately posterior to the uterus (Figure 1e). The oviduct loop is located 0.2–1.3 mm from the posterior end of the body. The ovary is long and has a ligament-like structure 12–78 μm in length (Figures 1f, 2b). The eggs exhibit the characteristic lemon-shaped (barrel-shaped) morphology typical of the genus *Trichuris*, with prominent bipolar plugs; the shell is thick, smooth, and finely porous. Their length is 64–74 μm and their width is 26–36 μm. Most of the eggs exhibited a smooth eggshell; however, some eggs presented an eggshell covered with numerous holes.

#### Males

**Figure 3:**
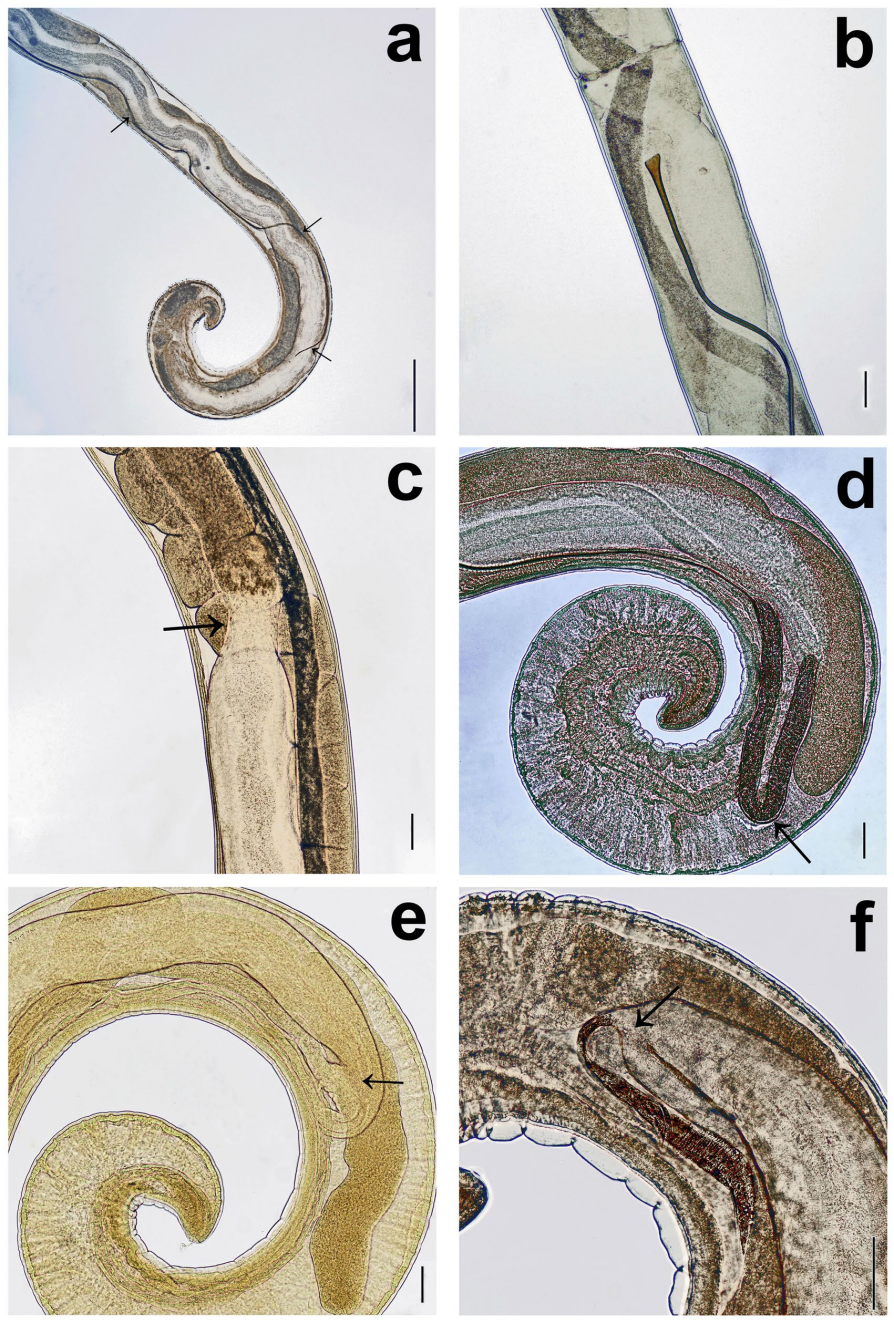
Males of *Trichuris tenuis*. **(a)** Long, slender, winding spicule (arrows). **(b)** The proximal portion of the spicule expands into a flare. **(c)** The junction between the ejaculatory duct and the vas efferens (arrow). **(d)** The cloaca forms a loop at the end of the body and turns anteriorly (arrow). **(e)** The intestine and the ejaculatory duct converge to form the cloaca (arrow). **(f)** The intestine and the ejaculatory duct converge to form the cloaca in another individual (arrow). Scale bars: **(a)** = 500 μm; **(b–f)** = 100 μm.

The total body length ranges from 26.3 to 43.4 mm, consisting of a slender anterior portion (13.8–25.6 mm) and a thicker posterior portion (8.8–18.4 mm). The width at the esophageal-intestinal junction is 116–258 μm, while the posterior portion measures 212–448 μm in width. The cuticular vesicle, or bacillary band, originates 378–667 μm from the anterior extremity and extends 1.5–1.8 mm posteriorly beyond its origin. The spicule is extremely slender and measures 4.7–5.6 mm in length (Figure 2c), with a shaft diameter of 4–10 μm; the proximal portion expands into a flare 23–46 μm wide, while the distal end is bluntly rounded (Figures 3a,b). The spicular sheath is narrow, sleeve-shaped, and aspinous, with a width of 11–20 μm. In all examined specimens, the spicule was fully retracted. The testis (7.3–13.6 mm long) originates 0.3–1.8 mm from the posterior end and is moderately convoluted throughout. It eventually straightens and narrows before transitioning into the vas efferens and into the ejaculatory duct. The vas efferens and the ejaculatory duct are distinguishable by their wall thickness; the former is thin-walled, whereas the latter possesses dense, muscular walls measuring 34–91 μm in width. A muscular constriction marks the junction of these two sections (Figures 2c, 3c). The cloaca is extremely long (5.0–6.7 mm) and does not extend directly posteriorly to the end of the body as in other species; instead, it turns forward and loops around other organs before joining the spicule pouch approximately at the level where it originates. The forward-passing tube and its junction with the spicular tube are very difficult to discern in most specimens. Depending on the specimen, the cloaca forms either a long (Figure 3d) or short loop (Figures 3e,f) and turns anteriorly at the junction of the intestine and ejaculatory duct (Figures 2a,b). Terminally, two caudal papillae (12–16 μm in diameter) are present, and the cuticle exhibits transverse striations.

### Molecular and phylogenetic studies

In total, 55 adult worms were characterized using three molecular markers, which yielded 30 unique sequences: two variants for 18S, 14 variants for ITSs, and 13 variants for COI. The observed variants were conserved across all three markers. Notably, COI exhibited the highest diversity, with sequence homology ranging from 98.7 to 99.9%. Detailed results of the BLAST analysis are presented in Table 2. The unique sequences generated during this study were deposited in the GenBank database under accession numbers PX842982-3 (18S), PX842984-97 (ITSs), and PX843293-306 (COI).

**Table 2:** Sequence analysis and BLAST results.

Gene	No. of seq	Sequence homology (%)	Sequence length (bp)	BLAST results^*^ (% query coverage; % identity)
18S	2	99.9	705	MW717988 (100; 99.7)
ITSs	14	99.2–99.8	1,054	HQ844233 (90; 85.6)
COI	13	98.7–99.9	1,201	MW750609 (35; 98.6)

^*^Alignments of all sequences for each genetic marker were used for BLAST analysis, and the nearest matches were identified based on BLAST scores.

**Figure 4:**
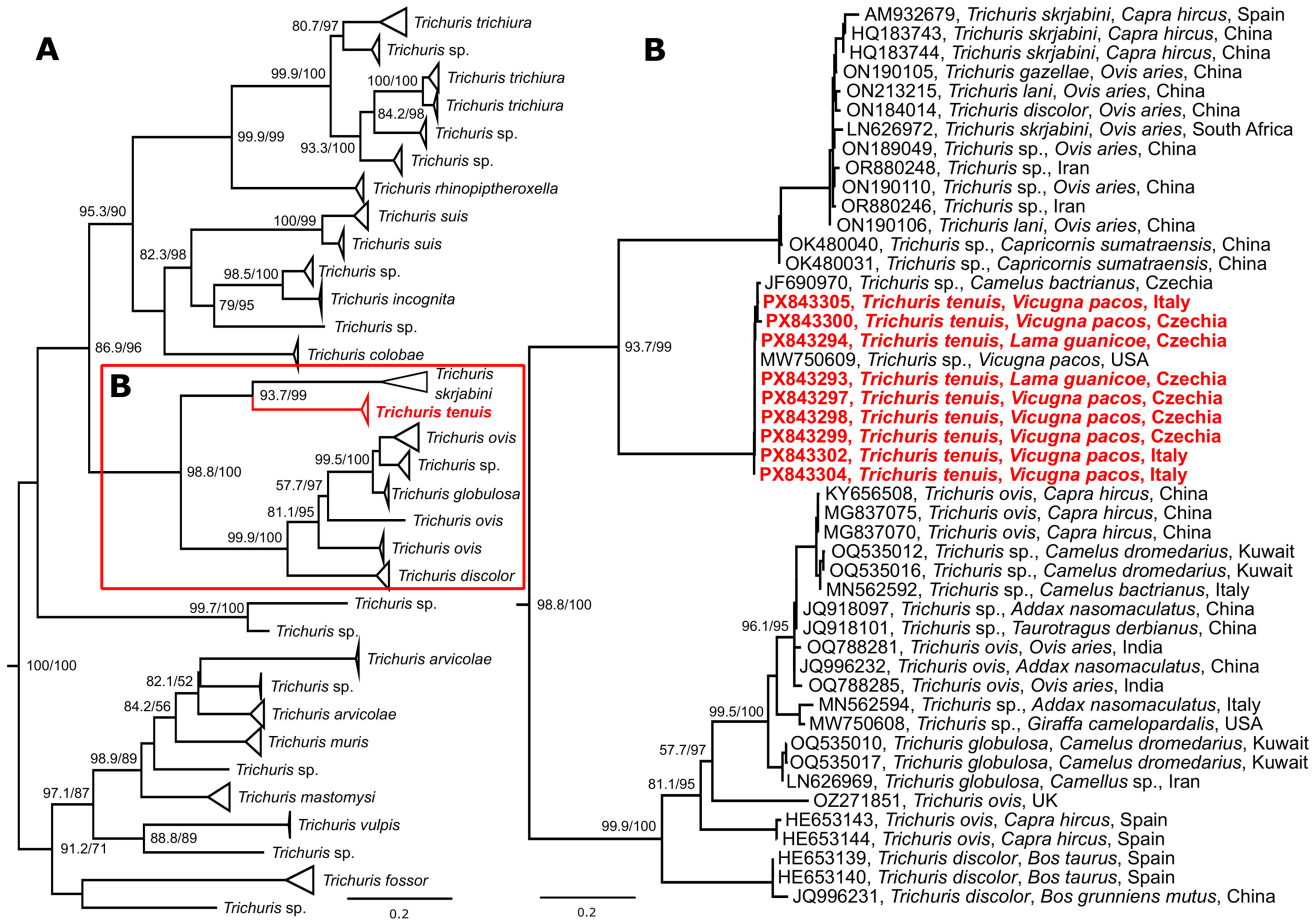
Schematic representation of a maximum likelihood phylogenetic tree based on the selected unique COI sequences of the genus *Trichuris* **(A)**, with a detailed view of herbivore-associated *Trichuris* spp. **(B)**. The final alignment length was 1,548 bp and included 130 sequences. The tree was constructed using the TIM + F + I + G4 evolutionary model. Two sequences of *Eucoleus aerophilus* used as an outgroup are not shown. The sequences of *Trichuris tenuis* generated in this study are marked in bold and red. The scale bar indicates the number of nucleotide substitutions per site. Bootstrap values (SH-aLRT/UFB) exceeding the 80/95 threshold are displayed. Sequences are labeled by accession number, species, host, and country of origin (if available).

**Figure 5:**
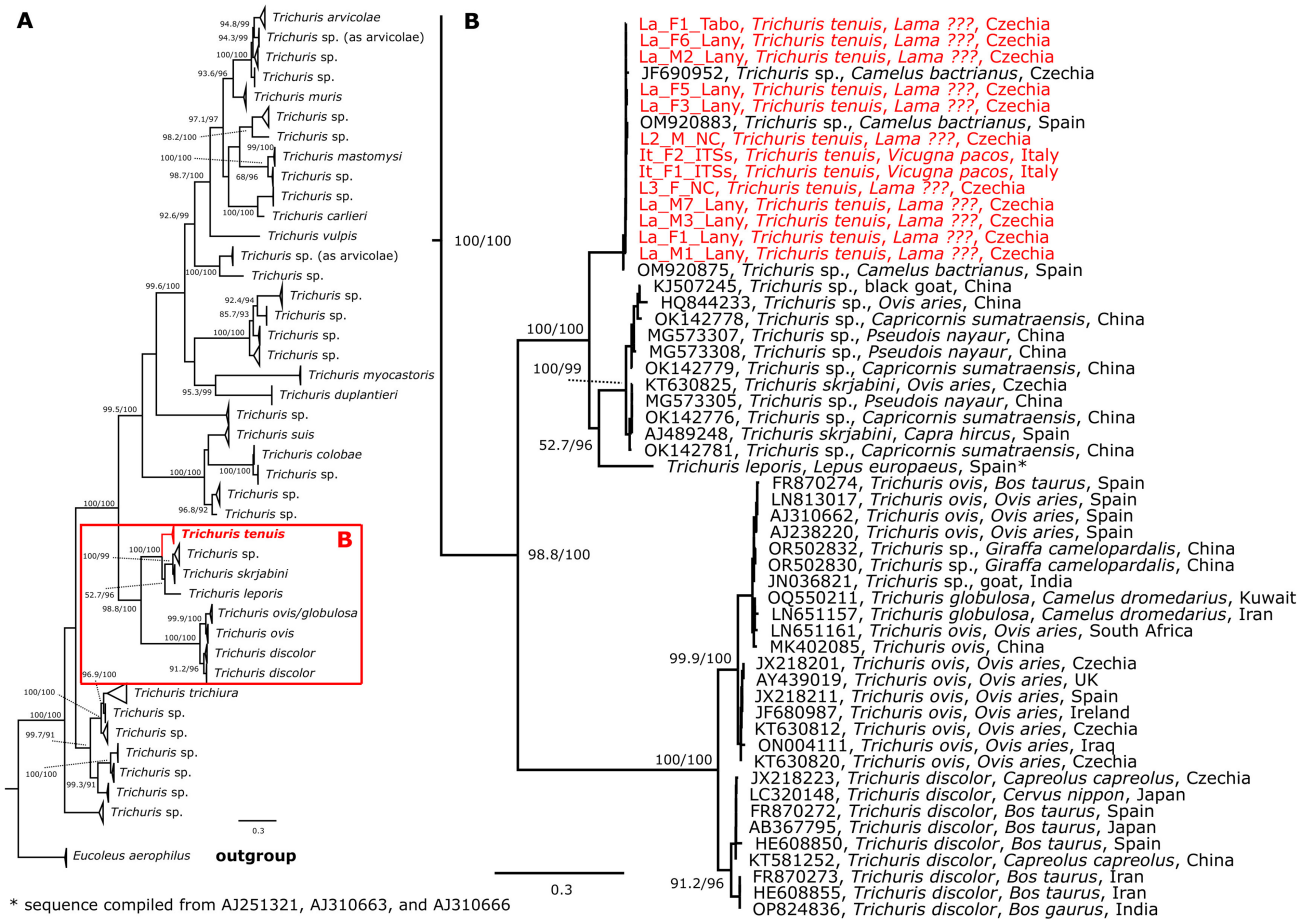
Schematic representation of a maximum likelihood phylogenetic tree based on the selected unique ITS (encompassing 18S rRNA, ITS1, 5.8 rRNA, ITS2, and 28S rRNA) sequences of the genus *Trichuris* **(A)**, with a detailed view of herbivore-associated *Trichuris* spp. **(B)**. The final alignment was prepared in two steps using the MAFFT algorithm. In the first step, sequences containing over 895 bp of the ITS1–ITS2 region were aligned. In the second step, the remainder of the selected sequences was added using the MAFFT_add function. The final alignment length was 2,461 bp and contained 218 sequences. The tree was constructed using the TPM2u + F + R3 evolutionary model. Three sequences of *Eucoleus aerophilus* were used as an outgroup. The sequences of *Trichuris tenuis* generated in this study are marked in bold and red. The scale bar indicates the number of nucleotide substitutions per site. Bootstrap values (SH-aLRT/UFB) exceeding the 80/95 threshold are displayed. Sequences are labeled by accession number, species, host, and country of origin (if available).

Three phylogenetic analyses were conducted in this study. *Trichuris* sequences primarily associated with herbivores formed a separate cluster. This cluster was further divided into two groups, one represented by species typically infecting ruminants *T. discolor*, *T. ovis* and *T. globulosa*, and second containing ruminant and also non-ruminant infecting species, represented by *T. tenuis*, *T. leporis* and *T. skrjabini*. In the COI phylogeny, two clades occupied a sister position to *T. tenuis*; these primarily comprised sequences obtained from *Trichuris* species infecting different ruminants in Asia. One of these sister clades contained identified sequences, predominantly *T. skrjabini* (Figure 4, Supplementary image 1). The topology of the ITS phylogeny was similar to that of the COI phylogeny, featuring a larger clade likely representing *T. skrjabini* and a second, distinct clade representing the lagomorph-infecting species *T. leporis* (Figure 5, Supplementary image 2). While the sequences of *T. tenuis* formed a distinct clade in the 18S phylogeny, this marker showed low resolution for differentiating individual species within the genus (Supplementary image 3). Across all three phylogenies, the *T. tenuis* clade included several previously published sequences, all designated as *Trichuris* sp. The most frequent host recorded for these parasites was the Bactrian camel (*Camelus bactrianus*), with additional occurrences in the alpaca (*V. pacos*), and northern giraffe (*Giraffa camelopardalis*). It is noteworthy that all sequences within the *T. tenuis* clade for every gene originated from specimens collected in zoos or on farms. Detailed phylogenies for all analyzed genes are provided in supplements.

## Discussion

Whipworms (Trichuridae) are common parasites of mammals, but their taxonomy remains complex and often controversial. In ruminants, Skrjabin et al. (43) and Knight (36) recognized 19 and 25 species, respectively, while Rickard and Bishop (19) listed 28 named taxa, although several of these are now considered synonyms (44–47). The lack of reference sequences for precisely identified nominal species, together with poorly understood host specificity for many taxa, further complicats molecular taxonomic approaches. Available phylogenies are providing evidence of species complexes within the genus *Trichuris*, further complicated by anticipated hybridisation events (12, 48–50). Yet, the names keep their value and integrative taxonomy, combining assessment of morphological data with sequence analyses represents a robust framework for understanding diversity and host specificity within the genus.

To date, 10 Trichuris species have been described within the family Camelinae (Table 3). Species identification has historically relied on morphological and morphometric characters, using host species as a diagnostic guide (51). In males, spicule length remains the primary diagnostic character (37, 38, 52), supplemented by the structure and shape of the spicule sheath, the morphology and arrangement of spicule sheath spines, the proximal and distal ends of the spicule, and the length and shape of the cloacal tube (37, 38, 49, 52–54).

**Table 3:** Species of the genus *Trichuris* recorded to date in herbivores in the family Camelinae.

Species	Host	Origin	Taxonomic identification method	Reference
*T. barbetonensis* Ortlepp, 1937	*Camelus dromedarius*	Iran	Morphology	Skrjabin et al. (43)
	*C. dromedarius*	Iran	Morphology	Borji et al. (68)
*T. cameli* Rudolphi, 1819	*C. dromedarius*, *C. bactrianus*	Europe, North America, India	Morphology	Skrjabin et al. (43)
	*C. dromedarius*	Iran	Morphology	Mirazayans and Halim (69)
*T. globulosa* (Linstow, 1901)	*C. dromedarius*	Kuwait	Morphology, molecular analysis	Henedi et al. (12)
	*C. dromedarius*	Iran	Morphology	Borji et al. (68)
	*C. dromedarius*	Iran	Morphology, molecular analysis	Callejón et al. (31)
	*C. dromedarius*	Egypt	Morphology, molecular analysis	Ismail et al. (13)
	*C. dromedarius*	Afghanistan	Morphology	Baruš et al. (70)
	*C. dromedarius*	Europe, Asia, Africa, former USSR	Morphology	Skrjabin et al. (43)
	*C. dromedarius*	Iran	Morphology	Mirazayans and Halim (69)
	*Camelidae*	Former USSR	Morphology	Gagarin (71)
*T. lama* Ezzat, 1945	*Lama glama*	Egypt	Morphology	Skrjabin et al. (43)
*T. lani* (Artjuch, 1948)	*C. bactrianus*	Former USSR	Morphology	Skrjabin et al. (43)
	*C. dromedarius*	Iran	Morphology	Mirazayans and Halim (69)
	*Camelidae*	Former USSR	Morphology	Gagarin (71)
*T. ovis* Abilgaard, 1795	*C. dromedarius*, *C. bactrianus*	Former USSR/cosmopolitan	Morphology	Skrjabin et al. (43)
	*Camelidae*	Former USSR	Morphology	Gagarin (71)
*T. skrjabini* Baskakov, 1924	*C. dromedarius*	Iran	Morphology	Mirazayans and Halim (69)
	*C. dromedarius*	United States	Morphology	Fowler (72)
	*C. dromedarius*	Australia	Morphology	Beveridge and Green (46)
	*C. dromedarius*, *C. bactrianus*	Former USSR	Morphology	Skrjabin et al. (43)
	*Camelidae*	Former USSR	Morphology	Gagarin (71)
*T. raoi* Alwar and Achutan, 1960	*C. dromedarius*	India	Morphology	Skrjabin et al., 1957 (43)
	*C. dromedarius*	Iran	Morphology	Mirazayans and Halim (69)
*T. tenuis* Chandler, 1930	*C. dromedarius*	United States	Morphology	Rickard and Bishop (19)
	*L. glama*	United States	Morphology	Rickard and Bishop (20)
	*L. glama*, *Vicugna vicugna*	Argentina	Morphology	Cafrune et al. (62)
	*C. dromedarius*	Iran	Morphology	Mirazayans and Halim (69)
	*Lama guanicoe*	Argentina	Morphology	Beldomenico et al. (73)
	*Vicugna pacos*, *L. glama*	United States	Morphology	Fowler (72)
	*V. pacos*	Australia	Morphology	Rashid et al. (74)
	*L. glama*	United Kingdom	Morphology	Welchman et al. (75)
	*V. pacos*	Peru	Morphology	Casas et al. (76)
	*C. dromedarius*	United States	Morphology	Skrjabin et al. (43)
*T. vulpis* (Froelich, 1798)	*C. dromedarius*	Iran	Morphology	Mirazayans and Halim (69)

*T. tenuis* is morphologically well-characterized, largely owing to the detailed descriptions of Chandler (38), from *C. dromedarius* and Rickard and Bishop (19) from *L. glama*. These authors emphasized that the primary diagnostic feature of this species is not the spicule length, as in many congeners, but the length of the male cloacal canal. Sarwar (47) reported *T. tenuis* also from goats in Cyprus, but his measurements are inconsistent with the species; notably, the author failed to report the exceptionally long cloaca in males, a main characteristic of *T. tenuis*.

The SAC trichurids in our study are visibly smaller and thinner than those of ruminant, especially compared to whipworm species such as *T. discolor* or *T. ovis*. Except for minor details, the morphological characteristics of *T. tenuis* males and females in our study were consistent with the descriptions of both Chandler (38) and Rickard and Bishop (19). In line with Chandler (38), our measurements indicated that the posterior portion of males is long and slender.

Of note, according to our observations, in males, the cloaca connects to the spicular tube slightly posteriorly. In contrast, Chandler (38) reported that “the cloaca is recurved at its origin and joins the spicular tube far anteriorly,” while Rickard and Bishop (19) found that the cloaca connects to the spicular tube anterior to, or at the point of origin of, the cloaca. We suggest that the point of connection between the cloacal tube and the spicular tube may vary, occurring either anteriorly or posteriorly relative to the cloaca’s origin. Additionally, our description of the testes also differs slightly from that of Chandler (38), as we did not observe them as forming a series of squarish compartments. However, based on our observations, we agree that the testes form sharp folds along their entire length.

All *T. tenuis* female specimens have a unique egg chamber, characterized as a distinct bulge located approximately in the mid-portion of the vagina. Although the vagina of this species is strongly convoluted, the middle section expands to form a chamber 122–375 μm wide (Figures 1d, 2a). A similar structure, termed an egg chamber, was described by Callejón et al. (31) in *T. globulosa*; however, unlike the structure observed here, it does not form a vaginal bulge, but instead represents an egg-filled segment of the vaginal loop.

Extensive overlap exists in measurements and biometrics among individuals of the genus *Trichuris*, including spicule length (31, 49, 55–57), and some morphological features may not be apparent in all individuals. For example, in species in which the female exhibits an everted vulva, this characteristic may remain obscured until the release of the first egg (*T. discolor*—our observation).

In the present study, three genetic markers, both nuclear and mitochondrial, were used to characterize *T. tenuis*. Our phylogenetic data showed that 18S is too conserved as a marker and cannot resolve detailed relationships of the genus, as seen in other organisms (58–60). Conversely, ITSs and COI showed much detailed resolution into well-supported clades and seem to be more reliable markers for the phylogeny of *Trichuris* spp. However, without sufficient knowledge about intraspecific variability it is impossible to differentiate between said variability and potential cryptic species.

It is noteworthy, that all sequences of *T. tenuis* available in the GenBank (including those originating from this study) come from captive animals from zoos or farms, thus the original host of this whipworm species remains in the mist. Old and New World camelids both originate in North America, yet both groups have diverged more than 15 mil years ago (61). Sequences of *T. tenuis* obtained from captive lamas in the North America and those from lamas and camels in Europe are very similar if not identical and it is very unlikely that *T. tenuis* survived unchanged the divergence of camelids. Rather, the parasite has probably infected either lamas in the New World or camels in the Old World originally and later adapted to the other species after they became mixed in captive settings. In Argentina, *T. tenuis* was morphologically identified in semi-captive vicuñas (62), suggesting the presence of the parasite also in wild populations of SAC. Interestingly, limited paleo-parasitological data indicate, that *T. tenuis* might have appeared in SAC after the arrival of Europeans into South America (63). Only future studies, focusing on wild population of both groups of camelids could help resolve the original parasite–host association for *T. tenuis* and real diversity and evolution of trichurid nematodes in these hosts.

In conclusion, we have combined morphological and phylogenetic analyses to characterize a whipworm species associated with domesticated camelids and propose this monophyletic taxon to be referred to as *T. tenuis*. Since all the data regarding *T. tenuis* comes from captive camelids outside their native geographic ranges, the original host of *T. tenuis* remains unclear. Nevertheless, the story of whipworms of captive camelids demonstrates the role which domestication and globalization can play in distribution of parasites.

## Data Availability

Sequences generated here are deposited in the NCBI Nucleotide database under accession numbers PX842982-3 (18S), PX842984-97 (ITSs), and PX843293-306 (COI).
