# Supplementary material for: Molecular and morphological characteristics of Trichuris tenuis from South American Camelids bred in Europe
Source: Front Vet Sci. 2026 May 7;13:1832113. doi: 10.3389/fvets.2026.1832113 (PMC13189723; doi:10.3389/fvets.2026.1832113)
Supplement: Supplementary file 1 [file image_1.pdf]

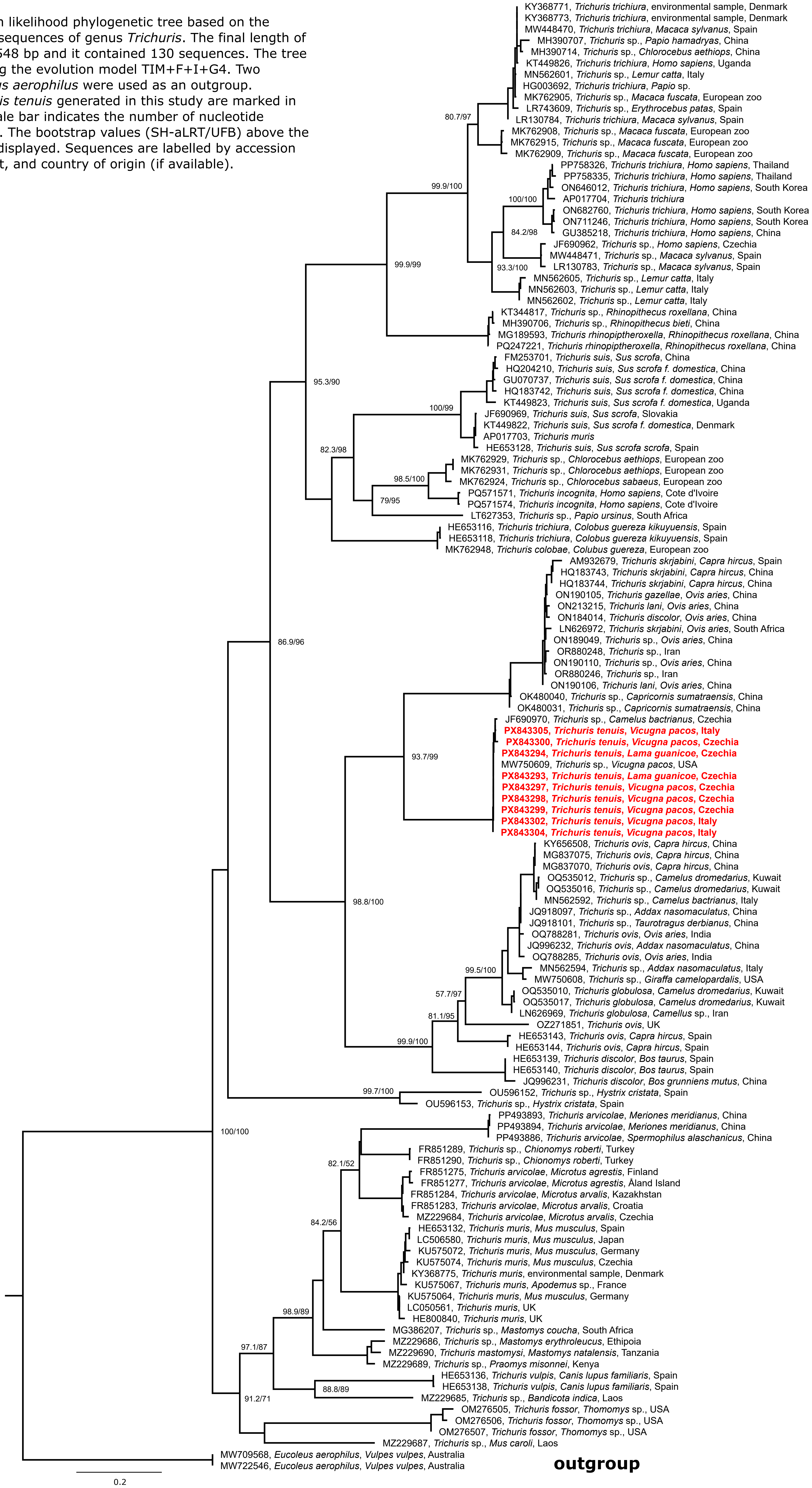

Figure S1: Maximum likelihood phylogenetic tree based on the selected unique *COI* sequences of genus *Trichuris*. The final length of the alignment was 1548 bp and it contained 130 sequences. The tree was constructed using the evolution model TIM+F+I+G4. Two sequences of *Eucoleus aerophilus* were used as an outgroup. Sequences of *Trichuris tenuis* generated in this study are marked in bold and red. The scale bar indicates the number of nucleotide substitutions per site. The bootstrap values (SH-aLRT/UFB) above the 80/95 threshold are displayed. Sequences are labelled by accession number, species, host, and country of origin (if available).
