# Supplementary material for: Molecular and morphological characteristics of Trichuris tenuis from South American Camelids bred in Europe
Source: Front Vet Sci. 2026 May 7;13:1832113. doi: 10.3389/fvets.2026.1832113 (PMC13189723; doi:10.3389/fvets.2026.1832113)

Figure S2: Maximum likelihood phylogenetic tree based on the selected unique ITSs sequences of genus *Trichuris* (comprising of 18S rRNA, ITS1, 5.8 rRNA, ITS2, and 28S).The alignment was prepared in two steps using MAFFT algorithm. In the first step, sequences containing at least ITS1-ITS2 region that were longer than 895 bp were aligned. In the second step, the reminder of selected sequences were added using the MAFFT_add function. The final length of the alignment was 2461 bp and it contained 218 sequences. The tree was constructed using the evolution model TPM2u+F+R3. Three sequences of *Eucoleus aerophilus* were used as an outgroup. Sequences of *Trichuris tenuis* generated in this study are marked in bold and red. The scale bar indicates the number of nucleotide substitutions per site. The bootstrap values (SH-aLRT/UFB) above the 80/95 threshold are displayed. Sequences are labelled by accession number, species, host, and country of origin (if available).

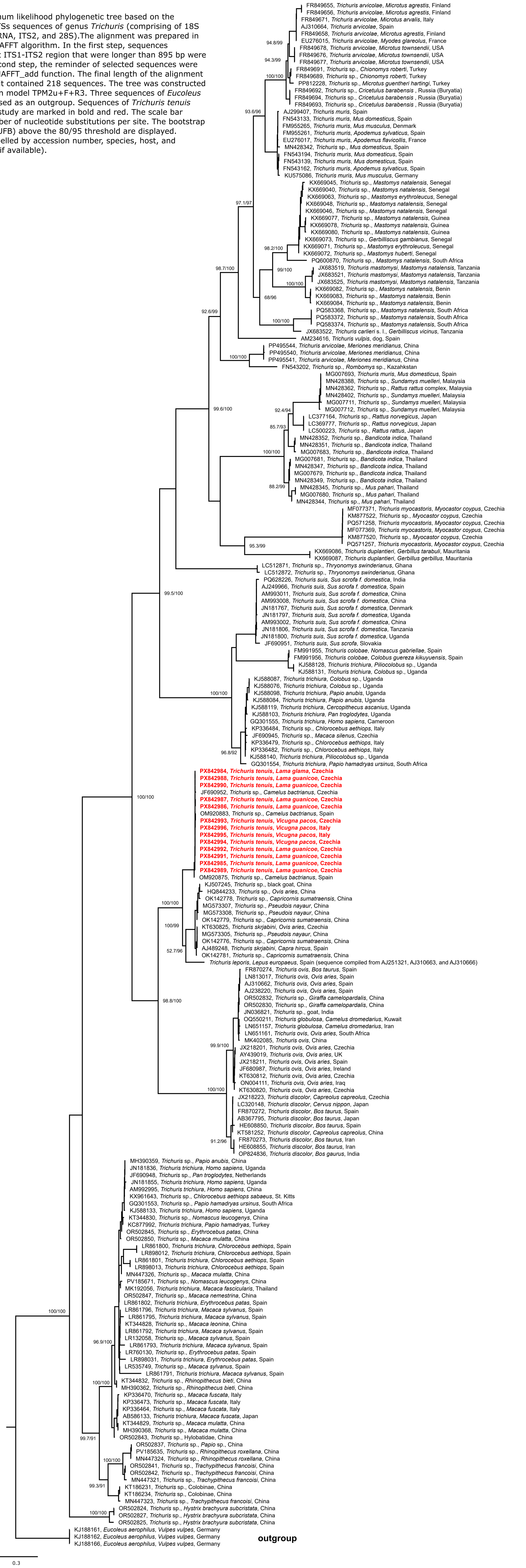

Supplement: Supplementary file 2 [file image_2.pdf]
