# Supplementary material for: Molecular and morphological characteristics of Trichuris tenuis from South American Camelids bred in Europe
Source: Front Vet Sci. 2026 May 7;13:1832113. doi: 10.3389/fvets.2026.1832113 (PMC13189723; doi:10.3389/fvets.2026.1832113)
Supplement: Supplementary file 4 [file table_1.pdf]

S1-Table: **Biometrical parameters of *Trichuris tenuis* isolated from South American Camelids from three farms and one Zoo.**

		Geographical location															
		F1CZ				F2CZ				F3It				Tábor Zoo			
**Females**		**n**	**mean**	**min-max**	**sd**	**n**	**mean**	**min-max**	**sd**	**n**	**mean**	**min-max**	**sd**	**n**	**mean**	**min-max**	**sd**
Total length	µm	10	30756	28630-33700	1793	5*	34362	28521-40534	4346	1*	43562	43562	0	1*	30152	30152	0
Length of anterior portion	µm	10	21231	18430-25640	2326	5*	25716	21546-32098	3896	1*	31658	31658	0	1*	20577	20577	0
Diameter of anterior part	µm	10	81	56-95	13	16	104	71-131	19	2*	111	98-124	18	2	136	122-149	19
Diameter at junction of esophagus and intestine	µm	10	159	103-273	46	16	222	180-256	26	2*	285	238-277	28	2	224	198-250	37
Length of posterior portion	µm	10	9525	7750-11380	1337	16	9104	7285-11090	1	6	9521	4755-17126	4441	2	10479	9548-11409	1316
Diameter of posterior portion	µm	10	442	344-687	96	16	624	472-794	90	6	668	516-968	171	2	653	490-816	231
Distance from anterior end to level of bacillary band	µm	4**	353	124-756	278	6**	517	332-804	183	6****				0*			
Distance bacillary band extend along anterior portion	µm	4**	1357	540-1931	603	6**	1279	773-1652	298	6****				0*			
Intervals between anterior striations	µm	10	7	5-10	1	16	9	5-11	1	6****				2**			
Intervals between posterior striations	µm	10	3	3-4	1	16	4,9	4-6	0,9	6****				2**			
Length of vagina	µm	10	2704	1958-3767	520	16	2339	1104-3210	455	6****				2	3023	2554-3493	664
Length of vulva	µm	8**	19	10-30	6	16	24	10-36	13	6****				2	21	17-25	6
Width of vulva	µm	8**	8	5-11	2	16	8	4-13	6	6****				2	4	4	0
Distance egg chamber from vulva	µm	10	1170	1050-1280	74	16	1165	873-1990	302	6****				2	1266	1230-1302	51
Length of egg chamber	µm	10	227	189-258	26	16	191	144-240	35	6****				2	183	159-206	33
Width of egg chamber	µm	10	166	122-196	20	16	255	188-375	51	6****				2	236	201-271	49
Length of uterus	µm	10	5078	3782-6367	737	16	5943	4076-7854	1156	6****				2	6618	6340-6896	393
Width of uterus	µm	10	372	260-422	53	16	456	208-538	89	6****				2	631	524-738	151
Distance from loop of oviduct to posterior end of body	µm	10	784	207-1261	429	16	733	373-1208	255	6****				2	397	335-459	88
Distance ovary from posterior end of body	µm	10	126	77-201	43	16	152	79-266	49	6****				2	132	107-158	36
Length of rectum	µm	10	203	128-261	50	16	199	132-274	41	6****				2	272	172-373	142
Length of ovary appendix	µm	5**	62	40-78	15	12**	30	12-48	13	6****				0**			
Length of egg	µm	10	67	65-70	2	15***	69	67-74	3	3**	73	67-77	5	2	64	61-67	4
Width of egg	µm	10	31	26-35	3	15***	33	30-36	2	3**	35	34-37	1	2	34	32-36	3
**Males**		**n**	**mean**	**min-max**	**sd**	**n**	**mean**	**min-max**	**sd**	**n**	**mean**	**min-max**	**sd**	**n**	**mean**	**min-max**	**sd**
Total length	µm	10	31690	26301-41700	4889	3*	32898	27495-40329	6653	2*	42193	41011-43375	1672				
Length of anterior portion	µm	10	16272	13816-25710	5942	3*	20029	15915-25601	5005	2*	24952	24883-25020	97				
Diameter of anterior part	µm	10	90	45-113	20	8	112	71-167	4	6	124	109-140	21				
Diameter at junction of esophagus and intestine	µm	10	147	116-222	35	8	217	187-258	23	6	207	202-211	6				
Length of posterior portion	µm	10	12674	8750-15990	2261	8	12408	8710-16287	2903	6	13094	9207-18355	3780				
Diameter of posterior part	µm	10	282	212-387	77	8	386	286-448	23	6	348	312-377	21				
Distance from anterior end to level of bacillary band	µm	4**	708	658-826	167	2**	465	378-450	51	6****							
Distance bacillary band extend along anterior portion	µm	4**	1670	1285-1843	271	2**	1547	1498-1596	69	6****							
Intervals between anterior striations	µm	9	8	6-11	2	8	9	6_11	2	6****							
Intervals between posterior striations	µm	7	3	2-4	1	8	5	4-7	1	6****							
Width of spicule sheath	µm	10**				8	15	11-20	3	6****							
Length of spicule	µm	1**	4836	4836	0	8	5266	4734-5620	366	6****							
Width of proximal end of spicule	µm	2**	28	28	0	8	31	23-46	8	6****							
Diameter of spicule	µm	2**	8.60	8-9	0.5	8	6	4-10	2	6****							
Length of vas efferens	µm	10**				8	983	836-1303	153	6****							
Length of vas deferens	µm	10**				8	3151	2121-4121	735	6****							
Length of ejaculatory duct	µm	10**				7**	7339	5486-9173	1383	6****							
Length of constriction between vas deferens and ejaculatc	µm	10**				8	121	79-152	23	6****							
Width of ejaculatory duct wall	µm	10**				8	71	34-91	23	6****							
Length of cloaca	µm	10**				7**	6191	5031-6707	712	6****							
Length of testis	µm	10**				7**	10570	7341-13595	2651	6****							
Distance testis from posterior end	µm	3**	1195	997-1587	339	7**	995	334-1761	687	6****							
Diameter of caudal papilla	µm	10**				8	13	12-16	1	6****							
Height of caudal papilla	µm	10**				8	8	7-14	2	6****							

* Other specimens from this locality had the anterior portion detached.

** The mentioned structures were not visible in all specimens.

*** One specimen was a young female; eggs were present only in the uterus, not in the vagina (53.1 × 30.7 µm).

**** Most of the posterior end was used for genetic analysis (Italy).
